# Bacterial Diversity Stabilizes Community Productivity

**DOI:** 10.1371/journal.pone.0034517

**Published:** 2012-03-28

**Authors:** Nico Eisenhauer, Stefan Scheu, Alexandre Jousset

**Affiliations:** 1 Georg August University Göttingen, J. F. Blumenbach Institute of Zoology and Anthropology, Göttingen, Germany; 2 University of Minnesota, Department of Forest Resources, St. Paul, Minnesota, United States of America; Uppsala University, Sweden

## Abstract

**Background:**

Stability is a crucial ecosystem feature gaining particular importance in face of increasing anthropogenic stressors. Biodiversity is considered to be a driving biotic force maintaining stability, and in this study we investigate how different indices of biodiversity affect the stability of communities in varied abiotic (composition of available resources) and biotic (invasion) contexts.

**Methodology/Principal Findings:**

We set up microbial microcosms to study the effects of genotypic diversity on the reliability of community productivity, defined as the inverse of the coefficient of variation of across-treatment productivity, in different environmental contexts. We established a bacterial diversity gradient ranging from 1 to 8 *Pseudomonas fluorescens* genotypes and grew the communities in different resource environments or in the presence of model invasive species. Biodiversity significantly stabilized community productivity across treatments in both experiments. Path analyses revealed that different aspects of diversity determined stability: genotypic richness stabilized community productivity across resource environments, whereas functional diversity determined stability when subjected to invasion.

**Conclusions/Significance:**

Biodiversity increases the stability of microbial communities against both biotic and abiotic environmental perturbations. Depending on stressor type, varying aspects of biodiversity contribute to the stability of ecosystem functions. The results suggest that both genetic and functional diversity need to be preserved to ensure buffering of communities against abiotic and biotic stresses.

## Introduction

Human activities are affecting the functioning of virtually all Earth's ecosystems *via* multiple and exacerbating environmental changes [1]. This evoked a scientific quest for stabilizing mechanisms within ecosystems and unifying across-ecosystem theories [2]–[6]. Biodiversity is contentiously discussed as one biotic ecosystem property determining stability and may thus be essential for human well-being [5], [7]. However, varying definitions of stability as well as the underrepresentation of certain study systems and stability measures, and differences in experimental designs complicate this discussion [3], [5], [8].

Though inconsistent reports still fan the ongoing debate [2], evidence accumulates that biodiversity significantly determines major facets of ecosystem stability, such as temporal [9] and spatial variability [10], resistance against perturbations [11] and invasions [12], resilience [13] and reliability [14]. The underlying notion is that diverse communities host a variety of life strategies that are able to respond differentially to environmental perturbations and maintain ecosystem functioning through a plethora of traits [3], [9], [15]. This means that asynchrony in species' responses to environmental fluctuations due to niche differences stabilize ecosystem functions at high diversity [6], [16]–[18]. This explanation may not only be crucial for the stability of ecosystem functions in response to temporal fluctuations but also to spatial fluctuations or varied environmental contexts, such as differences in resource availability/composition or when affected by biotic invasion. In fact, spatial and temporal stability are closely interrelated [19].

Microorganisms represent the functional backbone of virtually any ecosystem [20], [21], and it is essential to understand their response under changing abiotic and biotic conditions. Therefore, diversity–stability relationships in microbial communities need closer consideration [4], [14], [22]–[24]. Here we address this issue by considering various stability measures of microbial productivity as a function of genotypic richness and functional diversity, two of the most prominent indices of biodiversity. Recent research stressed that different aspects of diversity (e.g., species richness, functional diversity and phylogenetic diversity) are responsible for ecosystem functioning [25]–[27], and we propose that this also applies to ecosystem stability. Phylogenetic and/or functional diversity may better predict ecosystem functioning than species richness *per se* [26], [28]. In particular functional diversity of communities may be a major driver of their performance [26], [27] and stability, while species richness has been manipulated in the vast majority of previous studies [29]. We thus investigated the impacts of genotypic richness and functional diversity of bacterial communities in the present study.

An important feature of communities is the reliability [14], [30] or predictability [22], [31] of functioning, the “probability that a system (specific community) provides a consistent level of functioning” [14] after a certain amount of time, i.e., a consistent level of functioning in varied abiotic or biotic contexts. In contrast to temporal stability, reliability is commonly measured as the across-treatment variation in ecosystem functioning [30]–[32]. This measure can thus be used to investigate the stability of ecosystem functioning of a given community in varied abiotic and biotic contexts. To investigate the linkage between biodiversity and reliability in multiple contexts, we manipulated the diversity of *Pseudomonas fluorescens* communities. Diversity was expressed as genotypic richness and functional diversity (as defined by Petchey and Gaston [33]). We subjected the communities to varied resource environments and to invasion by functionally similar invaders, thereby simulating two of the most important human induced stressors of ecosystems [1]. We measured the reliability of the communities as the stability of productivity of a given bacterial community across the treatments. Since diverse communities are more likely to contain genotypes with different responses to varied abiotic and biotic contexts [6], [16], [17], we expected the productivity of genetically and functionally more diverse communities to be more stable.

## Materials and Methods

### Experimental set-up

#### Community composition

We built resident bacterial communities from eight *P. fluorescens* strains (CHA0, PF5, Q2-87, 1M1-96, MVP1-4, F113, Phl1C2 and Q8R1-96) as described previously [34]. Briefly, bacteria were grown in LB broth at 25°C, pelleted by centrifugation (10'000 g, 1 min), washed twice in 1% NaCl and adjusted to an OD_600_ of 0.5. All genotypes have a similar body size, and a comparable OD to CFU/mL ratio, and OD_600_ is a reasonable proxy for the biomass of the inoculated bacteria. We set up 21 different bacterial communities of different composition by randomly assembling these strains, establishing diversity levels of 1 (each of the eight monocultures once), 2 (eight different communities), 4 (four different communities) and 8 genotypes (one community replicated four times) (Table S1A). Each genotype was present in the same number of communities at each richness level. This bacterial diversity gradient allows testing general ecological questions, even if the bacterial diversity in natural systems may be higher, even at small spatial scales [35].

#### Varied resource experiment

We set up a resource richness gradient by combining the carbon sources glucose, mannose, fructose, sucrose and citrate in order to cover a gradient of 1 (each of the five single carbon sources), 2 (four combinations), 3 (four combinations) and 5 (all carbon sources together, replicated three times) carbon sources (Table S1B). We grew the bacteria in OS minimal medium, containing all minerals required for bacterial growth (Na_2_HPO_4_ 7.01 g l^−1^, KH_2_PO_4_ 6.8 g l^−1^, MgSO_4_ *7H_2_O 1.19 g l^−1^, (NH_4_)_2_SO_4_ 1.2 g l^−1^, CaCl_2_*7H_2_O 8.8*10^−2^ g l^−1^, FeSO_4_ *7H_2_O 7.0 _*_10^−3^ g l^−1^, (NH_4_)_6_Mo_7_O_24_ * 4 H_2_O 2.0*10^−4^ g l^−1^, Na-EDTA 2.5*10^−3^ g l^−1^. ZnSO_4_ *7H_2_O 1.11 g l^−1^, MnSO_4_×H_2_O 1.54*10^−3^ g l^−1^, CuSO_4_*5H_2_O 3.90*10^−4^ g l^−1^, Co(NO_3_)_2_×6 H_2_O 2.50*10^−4^ g l^−1^, Na_2_B_4_O_7_×10 H_2_O 1.80*10^−4^ g l^−1^, NiCl_2_*6H_2_O 1.30*10^−3^ g l^−1^;[36]), and supplemented with the above described resource combinations as sole carbon source (at uniform total concentration of 5 g l^−1^). Each bacterial community was grown in each of the resource treatments (24 bacterial treatments×16 resource treatments; 384 combinations in total).

#### Varied invader experiment

In order to test the effect of invasion on community performance, we selected the four resource treatments with three carbon sources (Table S1B; RC 10, 11, 12, 13). We grew *P. fluorescens* communities as described above, either alone or subjected to the invasion by *Serratia liquefaciens* MG1 or *Pseudomonas putida* IsoF, as described previously [34]. Both invaders were chromosomally tagged with Green Fluorescent Protein (GFP) using a mini-Tn7 transposon [37]. The invader species, two widespread rhizosphere bacteria [38], occupy similar ecological niches as the eight *P. fluorescens* strains, and the functional similarity between invaders and resident bacteria was expected to foster competitive interactions [28]. Increased competition for resources may result in increased interference by e.g., the production of toxins, and this may reduce community productivity. We set up a total of 288 combinations (24 bacterial×4 resource×3 invader treatments). This experiment was carried out at intermediate resource richness to take variation in resource composition among the treatments into account, while offering enough niche multidimensionality to allow resource complementarity.

#### Growth conditions

In both experiments, bacteria were grown in 384-well microtiter plates (Brand, Wertheim, Germany) at 25°C with agitation. All microcosms contained the same biomass at the beginning of the experiment (start OD_600_ = 0.05). After 24 h, we measured the optical density (OD_600_) as proxy for total community productivity.

### Calculations

Functional diversity of the community was calculated according to Petchey and Gaston [33] with the treedive() function in R 2.12 (R Development Core Team, Vienna, Austria). We used the growth (OD_600_) after 24 h at 25°C of each genotype on each of the five resources as phenotypic parameters. Functional diversity is a well established biodiversity index reflecting in our case the potential of mixed communities to use various substrates compared to monocultures. An index of 0 indicates the absence of complementarity in resource use, and higher values show an increased metabolic potential of the community. Stability (reliability) of community productivity was calculated as the inverse coefficient of variation (CV^−1^) in the varying treatments [3], [39] i.e., the reliability of productivity of a given bacterial community across different abiotic and biotic treatments.

### Statistical analyses

Data were log-transformed to meet the requirements of general linear models (GLMs; normality and homoscedasticity of errors), if necessary. We used sequential GLMs (type I sum of squares) to test in a hierarchical way the effects of genotypic richness and functional diversity [40] on the stability of community productivity. The sequential approach allows testing of correlated predictor variables, such as genotypic richness and functional diversity (*R* ^2^ = 0.88, *p*<0.0001; [41]), and avoids the overestimation of explained variance. *F*- and *p*-values for genotypic richness and functional diversity given in text refer to those where these variables were fitted first, allowing to compare their relative importance. In order to prevent the usage of pseudo-replicates (e.g., in case of the eight-genotype treatments; Table S1A), genotypic richness and functional diversity were tested against the variance explained by bacterial composition [41]. Thereby, we avoided overstating the effect of the eight-genotype communities, where the identical community was replicated four times. Bacterial communities with identical compositions received the same consecutive number to statistically separate biodiversity from composition effects (e.g., all eight-genotype communities received #21; see Table S1A). In addition, we performed separate GLMs with a reduced dataset of two and four genotype communities to test if bacterial diversity effects remained significant when the highest (8 genotypes) and the lowest (1 genotype) of the diversity gradient were removed, thereby investigating whether diversity effects were also significant at intermediate levels.

As a complement to the GLM approach used to investigate treatment effects and to account for the composition of bacterial communities, path analysis was employed to investigate if bacterial diversity effects on stability of community productivity were due to genotypic richness or functional diversity. Path analysis allows testing the strength of direct and indirect treatment effects by involving multiple causal pathways [42]. Hence, by using path analysis, we were able to test if bacterial genotype richness *per se* directly influenced stability of community productivity, or if productivity was indirectly stabilized by increasing functional diversity. In our path analyses arrows represent causal relationships or processes, while rectangles represent manipulated (genotypic richness) or measured variables (functional diversity and stability of community productivity). We hypothesized that genotypic richness (exogenous variable) influences stability (endogenous variable) either directly or indirectly through increasing bacterial functional diversity (endogenous variable). The adequacy of the model was determined via *χ^2^* tests and AIC (Akaike Information Criterion; [43]). Non-significant *χ^2^* tests (*p*>0.05) and low AIC values indicate that the model cannot be rejected as a potential explanation of the observed covariance structure [42]. Two separate models were tested for the varied resource experiment and the varied invader experiment. In contrast to the GLM approach, the path analyses did not consider bacterial community composition and therefore the overall significance of the two bacterial diversity measures, but focused on their relative importance. Path analysis was performed using Amos 5 (Amos Development Corporation, Crawfordville, FL, USA).

## Results

**Figure 1:**
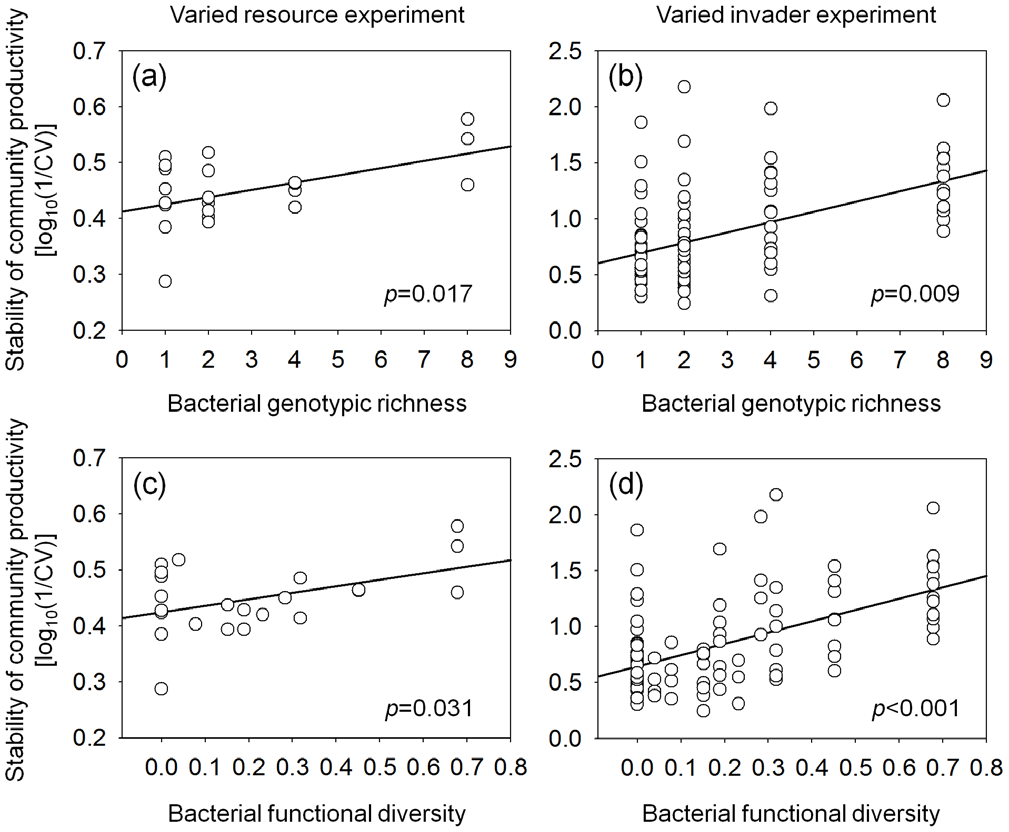
Stability of community productivity as affected by bacterial genotypic and functional diversity. Effects of bacterial genotypic (a, b) and functional diversity (c, d) on the stability of community productivity in varied resource environments (1/coefficient of variation of 14 resource treatments) (a, c) and invader treatments (no invader, *Pseudomonas putida* and *Serratia liquefaciens* as model invaders) (b, d). Each circle represents the stability of productivity of a given bacterial community in varied abiotic (a, c) or biotic environments (b, d).

Genotypic richness significantly stabilized community productivity in the varied resource experiment (*F_1,19_* = 6.90, *p* = 0.0166; Fig. 1A) and the varied invader experiment (*F_1,19_* = 8.42, *p* = 0.0092; Fig. 1B). Similarly, functional diversity significantly stabilized community productivity in the varied resource experiment (*F_1,19_* = 5.40, *p* = 0.0314; Fig. 1C) and the varied invader experiment (*F_1,19_* = 17.82, *p* = 0.0005; Fig. 1D). The increase in stability with functional diversity remained significant in the varied invader experiment using communities with intermediate diversity, i.e., with two and four genotypes only (*F_1,10_* = 6.63, *p* = 0.0276), but not in the varied resource experiment (*p*>0.3). In contrast, the increase in stability with genotypic richness was no longer significant in these analyses (all *p*>0.4). Data on invader performance will be presented in a separate study (N. Eisenhauer, W. Schulz, S. Scheu, A. Jousset, unpublished data).

**Figure 2:**
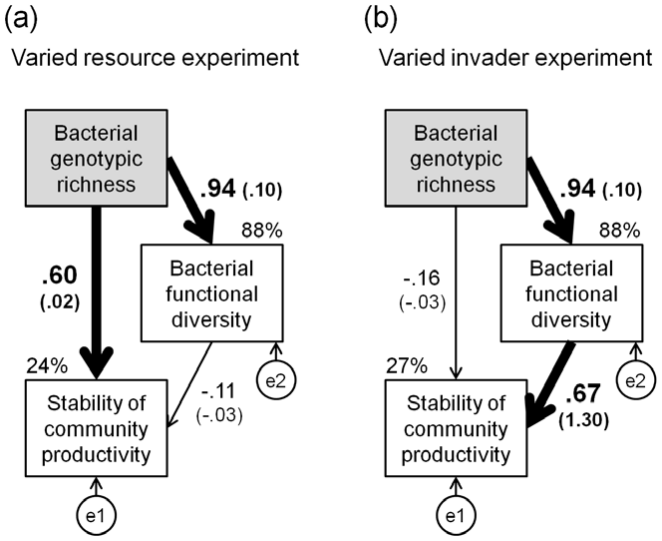
Relative importance of genotypic richness and functional diversity effects. Path analyses of direct and indirect (through increasing functional diversity) bacterial genotypic richness effects on stability of bacterial community productivity in the varied resource experiment (VRE) (a) and varied invader experiment (VIE) (b). While no *χ* ^2^-value could be calculated for the initial model for VRE (*AIC* 12.00), removing the arrow between functional diversity and stability resulted in a model with good fit to the data (*χ^2^_1,5_* = 0.05, *p* = 0.83, *AIC* 10.05). Similarly, no *χ^2^*-value could be calculated for the initial model for VIE (*AIC* 12.00), however, removing the non-significant path between genotypic richness and stability resulted in a model with good fit to the data (*χ^2^_1,5_* = 0.38, *p* = 0.54, *AIC* 10.38). The width of the arrows indicates the strength of the causal influence: bold arrows indicate significant standardized path coefficients (*P*<0.01; unstandardized path coefficients in brackets), whereas thin arrows indicate non-significant relationships (*P*>0.05). Exogenous variables are highlighted with grey rectangles, while endogenous variables are given in white. Values at the top corner of white rectangles are the variance of the respective variable explained by the model.

Path analysis indicated that bacterial diversity stabilized community productivity through an increasing number of genotypes in the varied resource environment (Fig. 2A), whereas stability significantly increased with increasing functional diversity in the varied invader experiment (Fig. 2B).

## Discussion

Biodiversity is a major predictor of the reliability of various communities including plants and microbes [3], [14], [31]. Our results indicate that biodiversity stabilizes the productivity of microbial communities in varied abiotic and biotic contexts for the first time using an across-replicate comparison of microbial productivity. Importantly, different biodiversity indices had to be considered to predict the stability of bacterial productivity. Genotypic richness, i.e., the number of genotypes present in a community, was the main driver of stability in varied resource environments, while functional diversity was closely related to the stability of communities subjected to invasion. This finding is surprising since genotypic richness and functional diversity were highly correlated in the present study, but the two diversity indices differed substantially in their explanatory power regarding stability in different environmental contexts. Although the mechanisms underlying the differential significance of genotypic richness and functional diversity cannot be uncovered with the design of the present study, the results provide guidelines for future experiments targeting these mechanisms.

The increase in the stability of community productivity with genotypic richness in varied resource environments is likely to be related to an insurance effect [18]: diverse communities are more likely to contain genotypes being able to use new and/or varied resources. If the increased growth of some genotypes is sufficient to compensate the lower growth of the ones that are unable to use the new resources, then aggregate community performance will remain stable across treatments [6], [16], [17]. This, however, implies that dominance between the strains will vary, and further experiments investigating the relative performance of the different genotypes are needed to understand the stability of community composition across treatments. Moreover, genotypic richness may have encompassed functional traits not captured by our functional diversity index [44].

Stability in the varied invader experiment was best explained by functional diversity. Niche preemption by functionally diverse resident communities reduces the success of invasive species [12], [45]. The higher stability of diverse communities in our experiments therefore suggests that niche preemption reduced the effect size of invaders. Hence, functional diversity, i.e., the diversity of functional traits involved in resource capture, likely was responsible for the increased invader resistance of (N. Eisenhauer, W. Schulz, A. Jousset, S. Scheu, unpublished data) and the decreased invader effect size within more diverse bacterial communities.

Our results indicate that communities of low diversity are likely to be more sensitive to environmental changes, while diverse communities more stably maintain their functioning. The importance of biodiversity for ecosystem functioning [4], [20], [22], [23] and stability [2], [3], [5] is well established. Adding to these findings, we showed that in varied abiotic and biotic contexts different aspects of microbial diversity account for stability. Genotypic richness increased the reliability of community productivity across different resource treatments. This indicates that species-rich communities may be buffered against changes in resource composition, and maintain their function in case of environmental changes or habitat degradation. In contrast, functional diversity was the best predictor for the reliability of community productivity when subjected to invasion. This suggests that functionally diverse communities cope better with new species, and maintain their functionality in presence of invaders. Different aspects of biodiversity of a given community therefore complement each other in warranting the stability of communities facing multiple stressors. Overall, the results suggest that for maximizing the stability of functions of natural communities facing multiple perturbations, such as those increased by anthropogenic activity, as many aspects of biodiversity as possible should be conserved.

## Supporting Information

Table S1 **Design of the experiments.**
(DOCX)
