# Supplementary material for: Bacterial Diversity Stabilizes Community Productivity
Source: PLoS One. 2012 Mar 28;7(3):e34517. doi: 10.1371/journal.pone.0034517 (PMC3314632; doi:10.1371/journal.pone.0034517)
Supplement: Table S1 — Design of the experiments. (DOCX) [file pone.0034517.s001.docx]

**Supporting Information**

**Table S1. Design of the experiments.** Composition of bacterial communities (a) and resource treatments (b). Bacterial communities and resource treatments with identical compositions were coded identically to statistically separate diversity from composition effects.

|  |  |  |  |  |  |  |  |  |  |  |  |  |
| --- | --- | --- | --- | --- | --- | --- | --- | --- | --- | --- | --- | --- |
| 1. Bacterial communities | | | |  |  |  |  |  |  |  |  |  |
|  | Sample | MVP1-4 | Q2-87 | CHA0 | F113 | Phl1C2 | PF-5 | 1M1-96 | Q8R1-96 | GR | BC | FD |
|  | 1 | 1 | 0 | 0 | 0 | 0 | 0 | 0 | 0 | 1 | 1 | 0.00 |
|  | 2 | 0 | 1 | 0 | 0 | 0 | 0 | 0 | 0 | 1 | 2 | 0.00 |
|  | 3 | 0 | 0 | 1 | 0 | 0 | 0 | 0 | 0 | 1 | 3 | 0.00 |
|  | 4 | 0 | 0 | 0 | 1 | 0 | 0 | 0 | 0 | 1 | 4 | 0.00 |
|  | 5 | 0 | 0 | 0 | 0 | 1 | 0 | 0 | 0 | 1 | 5 | 0.00 |
|  | 6 | 0 | 0 | 0 | 0 | 0 | 1 | 0 | 0 | 1 | 6 | 0.00 |
|  | 7 | 0 | 0 | 0 | 0 | 0 | 0 | 1 | 0 | 1 | 7 | 0.00 |
|  | 8 | 0 | 0 | 0 | 0 | 0 | 0 | 0 | 1 | 1 | 8 | 0.00 |
|  | 9 | 1 | 0 | 0 | 0 | 0 | 0 | 0 | 1 | 2 | 9 | 0.15 |
|  | 10 | 0 | 0 | 0 | 0 | 1 | 0 | 0 | 1 | 2 | 10 | 0.04 |
|  | 11 | 0 | 1 | 0 | 0 | 0 | 1 | 0 | 0 | 2 | 11 | 0.19 |
|  | 12 | 0 | 1 | 0 | 0 | 0 | 0 | 1 | 0 | 2 | 12 | 0.08 |
|  | 13 | 0 | 0 | 1 | 0 | 1 | 0 | 0 | 0 | 2 | 13 | 0.32 |
|  | 14 | 0 | 0 | 0 | 0 | 0 | 1 | 1 | 0 | 2 | 14 | 0.19 |
|  | 15 | 0 | 0 | 1 | 1 | 0 | 0 | 0 | 0 | 2 | 15 | 0.32 |
|  | 16 | 1 | 0 | 0 | 1 | 0 | 0 | 0 | 0 | 2 | 16 | 0.15 |
|  | 17 | 1 | 1 | 0 | 0 | 1 | 1 | 0 | 0 | 4 | 17 | 0.28 |
|  | 18 | 0 | 0 | 1 | 0 | 0 | 1 | 1 | 1 | 4 | 18 | 0.45 |
|  | 19 | 0 | 1 | 1 | 1 | 0 | 0 | 1 | 0 | 4 | 19 | 0.45 |
|  | 20 | 1 | 0 | 0 | 1 | 1 | 0 | 0 | 1 | 4 | 20 | 0.23 |
|  | 21 | 1 | 1 | 1 | 1 | 1 | 1 | 1 | 1 | 8 | 21 | 0.68 |
|  | 22 | 1 | 1 | 1 | 1 | 1 | 1 | 1 | 1 | 8 | 21 | 0.68 |
|  | 23 | 1 | 1 | 1 | 1 | 1 | 1 | 1 | 1 | 8 | 21 | 0.68 |
|  | 24 | 1 | 1 | 1 | 1 | 1 | 1 | 1 | 1 | 8 | 21 | 0.68 |
|  |  |  |  |  |  |  |  |  |  |  |  |  |
| 1. Resource treatments | | | |  |  |  |  |  |  |  |  |  |
|  | Sample | Glucose | Sucrose | Mannose | Citrate | Fructose |  |  |  | RR | RC |  |
|  | 1 | 1 | 0 | 0 | 0 | 0 |  |  |  | 1 | 1 |  |
|  | 2 | 0 | 1 | 0 | 0 | 0 |  |  |  | 1 | 2 |  |
|  | 3 | 0 | 0 | 1 | 0 | 0 |  |  |  | 1 | 3 |  |
|  | 4 | 0 | 0 | 0 | 1 | 0 |  |  |  | 1 | 4 |  |
|  | 5 | 0 | 0 | 0 | 0 | 1 |  |  |  | 1 | 5 |  |
|  | 6 | 1 | 0 | 0 | 0 | 1 |  |  |  | 2 | 6 |  |
|  | 7 | 1 | 1 | 0 | 0 | 0 |  |  |  | 2 | 7 |  |
|  | 8 | 0 | 1 | 1 | 0 | 0 |  |  |  | 2 | 8 |  |
|  | 9 | 0 | 0 | 1 | 1 | 0 |  |  |  | 2 | 9 |  |
|  | **10** | **1** | **0** | **1** | **0** | **1** |  |  |  | **3** | **10** |  |
|  | **11** | **0** | **0** | **1** | **1** | **1** |  |  |  | **3** | **11** |  |
|  | **12** | **0** | **1** | **0** | **1** | **1** |  |  |  | **3** | **12** |  |
|  | **13** | **1** | **1** | **0** | **1** | **0** |  |  |  | **3** | **13** |  |
|  | 14 | 1 | 1 | 1 | 1 | 1 |  |  |  | 5 | 14 |  |
|  | 15 | 1 | 1 | 1 | 1 | 1 |  |  |  | 5 | 14 |  |
|  | 16 | 1 | 1 | 1 | 1 | 1 |  |  |  | 5 | 14 |  |
|  |  |  |  |  |  |  |  |  |  |  |  |  |

GR = genotypic richness, BC = bacterial community composition, FD = functional diversity, RR = resource richness, RC = resource composition. RC treatments used in the invasion study are given in bold.
